# External Validation of a Novel Lung Injury Prevention Score for the Emergency Department

**DOI:** 10.5811/westjem.41994

**Published:** 2025-12-19

**Authors:** Michael S. Char, Chuan-Chin Huang, Adit A. Ginde, Peter C. Hou

**Affiliations:** * Lahey Hospital and Medical Center, Department of Emergency Medicine, Burlington, Massachusetts; † Brigham and Women’s Hospital, Division of Global Health Equity, Department of Medicine, Boston, Massachusetts; ‡ University of Colorado School of Medicine, Department of Emergency Medicine, Aurora, Colorado; § Brigham and Women’s Hospital, Division of Emergency Critical Care Medicine, Department of Emergency Medicine, Boston, Massachusetts

## Abstract

**Introduction:**

Despite numerous randomized controlled trials, lung protective ventilation and prone positioning remain the only therapies shown to have a survival benefit in acute respiratory distress syndrome (ARDS). A National Heart, Lung, and Blood Institute workshop on the future of clinical research in ARDS suggested that identification of at-risk patients earlier in their clinical course would allow implementation of prevention strategies and facilitate study of these interventions. To this end, the Lung Injury Prevention Score (LIPS) was derived and validated to identify patients at risk of developing ARDS upon hospital admission, and the Emergency Department Lung Injury Prevention Score (EDLIPS) was subsequently derived and internally validated. For this study, we sought to externally validate EDLIPS.

**Methods:**

We performed a validation study of EDLIPS, using data from a large, multicenter trial— the Vitamin D to Improve Outcomes by Leveraging Early Treatment (VIOLET) trial. After identifying patients who met VIOLET inclusion criteria while in the ED, variables comprising EDLIPS were extracted for each patient. We calculated area under the receiver operating characteristic curves (AUC) of EDLIPS for the VIOLET dataset.

**Results:**

We identified a total of 1,270 patients. The mean age was 56, and 55% were male. The incidence of ARDS was 8.1%. EDLIPS discriminated patients who developed ARDS from those who did not with an AUC of 0.786 (95% CI, 0.740–0.832), nearly identical to its performance in the original study, which yielded an AUC of 0.784 (95% CI, 0.748–0.820).

**Conclusion:**

We successfully validated a risk-prediction model for the identification of ED patients at risk for ARDS in a large cohort of critically ill patients. The development of ARDS prevention trials will involve collaboration with other clinical groups, such as emergency physicians, to enroll patients as early as possible in their clinical course. EDLIPS is the first tool of its kind to undergo external validation, and it can aid in the identification of ED patients at risk for the development of ARDS.

## INTRODUCTION

Acute respiratory distress syndrome (ARDS) is recognized as a major cause of morbidity and mortality in critically ill patients. Despite numerous randomized controlled trials, lung protective ventilation and prone positioning remain the only therapies shown to have a survival benefit.^1^–^6^ According to the “two-hit” model of ARDS development, a pre-ARDS state exists following initial lung injury.^7^ Interventions in this state could result in significant syndrome mitigation or prevention. Delayed recognition of at-risk patients likely contributed to failed prevention trials.^8^ The short interval between risk exposure and development of ARDS and the small percentage of patients at risk of ARDS who ultimately develop the syndrome make enrollment in preventative studies challenging.^9^–^10^ As noted by a National Heart, Lung, and Blood Institute (NHLBI) workshop on the future of clinical research in ARDS, identification of at-risk patients early in their clinical course would allow earlier implementation of prevention strategies and facilitate study of these interventions.^11^ To this end, the Lung Injury Prevention Score (LIPS) was derived and validated to identify patients at risk of developing ARDS upon hospital admission.^8^ This score was derived from a mixed population, with 78% of patients admitted from the emergency department (ED) and 22% of patients admitted following high-risk, elective surgery.^8^

Although ARDS is classically thought of as an intensive care unit (ICU) syndrome, it can develop as soon as several hours after initial ED presentation.^12^ When used in a prospective fashion to identify ED patients at high risk for ARDS, LIPS significantly underperformed.^5^ Given the heterogenous cohort used to initially derive LIPS, investigators have subsequently endeavored to identify patients at risk of developing ARDS from more specific cohorts.^13^–^15^ Kor et al performed a subgroup analysis of a large, multicenter observational study to develop a risk-prediction model for postoperative patients at risk of developing ARDS.^13^

The ED Lung Injury Prevention Score (EDLIPS) was derived from a sample of 4,361 ED patients for the identification of ED patients at risk for ARDS.^12^ Compared to LIPS, EDLIPS identifies patients at risk for ARDS from a more extensive index of presenting symptoms and predisposing conditions. Although EDLIPS performed similar to LIPS in the original derivation cohort, this risk-prediction model has not been externally validated.^12^ To address this, we used the Vitamin D to Improve Outcomes by Leveraging Early Treatment (VIOLET) trial cohort.^16^ In the current study, we validate EDLIPS in an independent sample of critically ill patients.

## METHODS

### Study Design

This was a validation study of EDLIPS, using data from a large, prospective, multicenter trial—the VIOLET trial.^16^–^17^ This was a secondary analysis of de-identified trial data from the VIOLET study, conducted under a prior single institutional review board-approved protocol. The present validation study was approved by the NHLBI Prevention and Early Treatment of Acute Lung Injury (PETAL) Network.

### Study Population

From April 2017—July 2018, 44 United States hospitals enrolled adult patients who were admitted to the ICU with at least one acute risk factor for death or lung injury that contributed to their need for ICU admission and who screened positive for vitamin D deficiency. Acute risk factors for death or lung injury included pneumonia, aspiration, smoke inhalation, lung contusion, pancreatitis, sepsis, shock, and mechanical ventilation for acute respiratory failure. The primary end point was 90-day, all-cause, all-location mortality. Notably, ARDS prevention was a main focus of the VIOLET trial, as it was designed to include patients at higher risk for ARDS. LIPS was calculated for each patient upon enrollment, and patients were prospectively monitored for the development of ARDS within the first seven days of hospitalization. ARDS was diagnosed according to the Berlin definition.^18^

**Population Health Research Capsule**

What do we already know about this issue?

*Acute respiratory distress syndrome (ARDS) is a major cause of morbidity and mortality with few effective therapies. Research has shifted toward ARDS prevention*.

What was the research question?

*Can the ED Lung Injury Prevention Score (EDLIPS) discriminate ED patients who develop ARDS from those who do not in an external cohort?*

What was the major finding of the study?

*EDLIPS successfully discriminated patients who developed ARDS from those who did not with an AUC of 0.786 (95% CI, 0.740–0.832)*

How does this improve population health?

*EDLIPS can aid in the identification of at-risk ED patients to support future ARDS trials and bedside interventions*.

### Study Variables

For this validation study, the above cohort was restricted to those patients who met VIOLET inclusion criteria while in the ED. Variables comprising EDLIPS were extracted from the trial database for each patient. We treated any missing data as an absent disease state or a normal variable, following the same methodology as the original EDLIPS study. Baseline characteristics, illness severity, and incidence of ARDS were also obtained from the database. The primary outcome was development of ARDS within seven days of hospital admission.

### Statistical Analysis

The primary analysis assessed the predictive capability of EDLIPS in distinguishing those patients who developed ARDS from those who did not. We performed statistical analysis using R, package vR.4.5.1 (The R Institute for Statistical Computing, Vienna, Austria). Discrimination of EDLIPS performance was evaluated through the calculation of area under the receiver operating characteristic curves (AUC). Additionally, we computed corresponding positive and negative predictive values and their associated 95% confidence intervals.

## RESULTS

We identified 1,270 patients meeting inclusion criteria in the VIOLET cohort and included them in this validation study. The mean age was 56, and 55% of patients were male. The mean Sequential Organ Failure Assessment score was 5, and 28-day all-cause, all-location mortality was 14.6%. The mean EDLIPS score was 6.80. The incidence of ARDS was 8.1% (Table 1).

**Figure 1:**
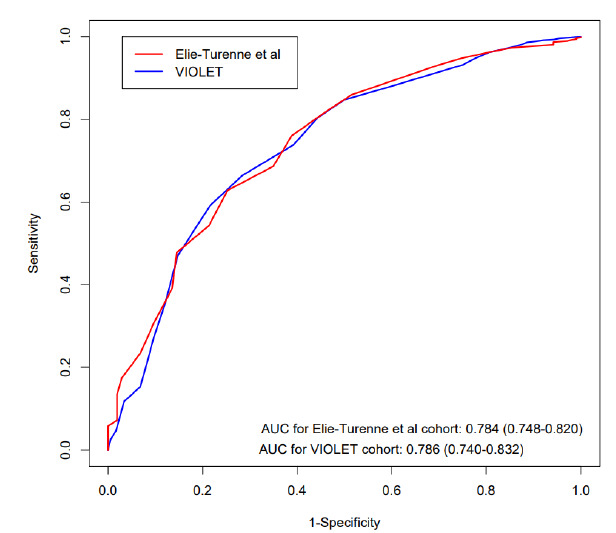
Area under the receiver operating characteristic curves comparing EDLIPS* performance in the original derivation cohort and the VIOLET** dataset with missing data considered absent disease state or a normal variable. *AUC*, area under receiver operating characteristic curve; *EDLIPS, ED Lung Injury Prevention Score; **VIOLET, Vitamin D to Improve Outcomes by Leveraging Early Treatment trial.

Of the patients identified, 757 had all variables needed to calculate EDLIPS. Most of the incomplete data were due to the absence of a serum albumin level or serum pH. EDLIPS discriminated VIOLET patients who developed ARDS from those who did not with an AUC of 0.786 (95% CI, 0.740–0.832), nearly identical to its performance in the original study, which yielded an AUC of 0.784 (95% CI, 0.748–0.820) (Figure 1). When we omitted missing data, the AUC remained unchanged (0.79, 95% CI, 0.73–0.83).

## DISCUSSION

Over the last decade, particularly in the wake of the COVID-19 pandemic, US hospitals have witnessed a dramatic increase in ED crowding and boarding. Peterson et al reported that the majority of increased volume was due to high-acuity patients.^19^ Increased ED boarding is associated with increased rate of hospital mortality and greater length of stay (LOS).^20^ Additionally, ED boarding of critically ill patients is associated with a variety of poor outcomes, including increased ICU LOS, prolonged mechanical ventilation, and higher mortality.^21^ Up to 7% of at-risk ED patients will develop ARDS, most within two days of presentation.^7^ Following trends toward early risk stratification and goal-directed therapy in other disease processes, emphasis has shifted toward early ARDS identification, with the goal of ARDS mitigation or prevention.^11^

In this external validation study, we confirmed the predictive capability of EDLIPS in distinguishing patients who developed ARDS from those who did not in a large, independent cohort of critically ill ED patients at risk for ARDS. Indeed, the predictive capability of EDLIPS remained robust, even when all missing data were excluded from analysis. This is the first time that EDLIPS has been validated in this fashion. The development of ARDS prevention trials will require tools to rapidly identify and enroll ED patients early in their clinical course. EDLIPS can aid in the identification and risk stratification of ED patients at risk of developing ARDS. Furthermore, EDLIPS may have a current role at the bedside. As ED patients who are admitted to the ICU spend prolonged time boarding in the ED, EDLIPS could be applied to identify high-risk patients for focused interventions, such as lung protective ventilation to avoid ventilator-associated lung injury.

## LIMITATIONS

The VIOLET study used the modern, Berlin definition of ARDS, whereas the original EDLIPS study utilized the 1994 American-European Consensus Conference (AECC) definition. As a result, there are several differences in the diagnostic criteria used in the two cohorts.^18^ A major change in the Berlin definition was removal of the term “acute lung injury” to describe mild forms of ARDS. Additionally, the Berlin definition removed the requirement of a pulmonary artery wedge pressure < 18 millimeters of mercury, refined chest radiograph criteria, and added a minimal positive end expiratory pressure requirement. Although the original EDLIPS cohort used the AECC definition, it incorporated two criteria that were ultimately included in the Berlin definition—timing of ARDS diagnosis and risk factors for ARDS. Using a dataset with ARDS identification and adjudication identical to the original EDLIPS study was prohibitive, especially given that the definition of ARDS changed in the decade between the two datasets. Notably, a study by Kim et al found that the original LIPS accurately predicted the development of ARDS as diagnosed by the Berlin definition, suggesting durability of these risk-prediction models.^22^

Although smaller than the original EDLIPS derivation cohort, the VIOLET cohort used in this study represents one of the largest available datasets of critically ill patients at risk for ARDS. Furthermore, the availability of large datasets investigating ARDS development as a primary outcome is severely limited. Since this work was a secondary analysis of trial data, our work is subject to the usual limitations of using data not collected specifically for this purpose.

The VIOLET study only enrolled patients after the clinician’s decision to admit the patient to the ICU, introducing the potential for selection bias and limiting generalizability. Additionally, no data were collected on ED LOS. Given that prolonged ED LOS is associated with worse outcomes in critically ill patients, ED LOS represents a potentially uncontrolled confounder. However, all patients in the cohort met inclusion in the ED with an average time from inclusion to randomization of < 7 hours. Now that the NHLBI PETAL Network has ended, future ARDS investigations will require new, large, multicentered datasets of ARDS patients with a greater focus on ED patients.

## CONCLUSION

We successfully validated a risk-prediction model for the identification of ED patients at risk for ARDS in a large cohort of critically ill patients. Further work is needed to assess efficacy in all comers to the ED. This is the first such tool to undergo external validation, and it can aid in the identification of at-risk ED patients to support future trials and bedside interventions.

## Supplementary Information



## Figures and Tables

**Table 1:** Demographic data and cohort characteristics of patients included in a study of the predictive capability of the Emergency Department Lung Injury Prevention Score (EDLIPS).

Characteristic	All (N = 1,270)	No ARDS (n = 1,167)	ARDS (n = 103)	*P*-values
Age (mean)	56	56	58	.35
Sex				
Male (%)	703 (55.4)	646 (55.4)	57 (55.3)	> .99
Female (%)	567 (44.6)	521 (44.6)	46 (44.7)	
Race and ethnicity				.04
Black (%)	279 (22.0)	261 (22.4)	18 (17.5)	
Non-Black Hispanic (%)	66 (5.2)	59 (5.1)	7 (6.8)	
Non-Hispanic White (%)	692 (54.5)	641 (54.9)	51 (49.5)	
Other (%)	36 (2.8)	35 (3.0)	1 (1.0)	
Not available (%)	197 (15.5)	171 (14.7)	26 (25.2)	
EDLIPS variables				
Aspiration (%)	63 (5.0)	45 (3.9)	18 (17.5)	< .001
Pneumonia (%)	456 (35.9)	397 (34.0)	59 (57.3)	< .001
Sepsis (%)	441 (34.7)	411 (35.2)	30 (29.1)	.26
Shock (%)	451 (35.5)	420 (36.0)	31 (30.1)	.28
Lung contusion (%)	40 (3.1)	39 (3.3)	1 (1.0)	.31
Smoke inhalation (%)	2 (0.20)	2 (0.20)	0 (0.0)	> .99
Long bone fracture (%)	19 (1.5)	18 (1.5)	1 (1.0)	.97
Brain injury (%)	21 (1.7)	18 (1.5)	3 (2.90)	.52
Cardiac surgery (%)	9 (0.7)	9 (0.8)	0 (0.0)	.78
Aortic surgery (%)	3 (0.2)	3 (0.3)	0 (0.0)	> .99
Acute abdomen (%)	14 (1.1)	12 (1.0)	2 (1.9)	.72
Diabetes mellitus (%)	139 (10.9)	131 (11.2)	8 (7.8)	.36
Cirrhosis (%)	68 (5.4)	61 (5.2)	7 (6.8)	.65
Chemotherapy (%)	125 (9.8)	114 (9.8)	11 (10.7)	.90
Obesity (%)	471 (37.1)	420 (36.0)	51 (49.5)	< .001
Acidosis (%)	446 (35.1)	367 (31.4)	79 (76.7)	< .001
FiO_2_ > 0.35 (%)	618 (48.7)	516 (44.2)	102 (99)	<0.001
Albumin <3.5 (%)	942 (74.2)	864 (74.0)	78 (75.7)	.80
SpO_2_ <95% (%)	860 (67.7)	775 (66.4)	85 (82.5)	< .001
Illness severity				
SOFA score (mean)	5	5	9	< .001
Mechanical ventilation (%)	362 (28.5)	264 (22.6)	98 (95.1)	< .001
Vasopressor use at baseline (%)	403 (31.7)	357 (30.6)	46 (44.7)	< .001
EDLIPS score (mean)	6.80	6.57	9.38	< .001
28-day mortality (%)	185 (14.6)	149 (12.8)	36 (35.0)	< .001
Time from randomization to intent to admit to ICU (hours)	6.77	6.77	6.8	.93

P-values were calculated using the Student *t*-test for continuous variables; chi-square test for categorical variables.

*ARDS*, acute respiratory distress syndrome; *EDLIPS*, emergency department lung injury prevention score; *FiO_2_*, fraction of inspired oxygen*; ICU*, intensive care unit; *SOFA*, Sequential Organ Failure Assessment; *SpO_2_*, oxygen saturation.

## References

[1] Meade MO, Jacka MJ, Cook DJ (2004). Survey of interventions for the prevention and treatment of acute respiratory distress syndrome. Crit Care Med.

[2] (2002). Randomized placebo-controlled trial of lisofylline for early treatment of acute lung injury and acute respiratory distress syndrome. Crit Care Med.

[3] The ARDS Network Authors for the ARDS Network (2000). Ketoconazole for early treatment of acute lung injury and acute respiratory distress syndrome, a randomized controlled trial. JAMA.

[4] Zeiher BG, Artigas A, Vincent JL (2004). Neutrophil elastase inhibition in acute lung injury: results of the STRIVE study. Crit Care Med.

[5] Kor DJ, Carter RE, Park PK (2016). Effect of aspirin on development of ards in at-risk patients presenting to the emergency department: the LIPS-A randomized clinical trial. JAMA.

[6] ARDS Network (2000). Ventilation with lower tidal volumes as compared with traditional tidal volumes for acute lung injury and the acute respiratory distress syndrome. N Engl J Med.

[7] Hou PC, Elie-Turenne MC, Mitani A (2012). Towards prevention of acute lung injury: frequency and outcomes of emergency department patients at-risk – a multicenter cohort study. Int J Emerg Med.

[8] Gajic O, Dabbagh O, Park PK (2011). Early identification of patients at risk of acute lung injury. Am J Respir Crit Care Med.

[9] Ferguson ND, Frutos-Vivar F, Esteban A (2007). Clinical risk conditions for acute lung injury in the intensive care unit and hospital ward: a prospective observational study. Crit Care.

[10] Soto GJ, Kor DJ, Park PK (2016). Lung injury prediction score in hospitalized patients at risk of acute respiratory distress syndrome. Crit Care Med.

[11] Spragg RG, Bernard GR, Checkley W (2010). Beyond mortality. Am J Respir Crit Care Med.

[12] Elie-Turenne MC, Hou PC, Mitani A (2012). Lung injury prediction score for the emergency department: first step towards prevention in patients at risk. Int J Emerg Med.

[13] Kor DJ, Lingineni RK, Gajic O (2014). Predicting risk of postoperative lung injury in high-risk surgical patients: a multicenter cohort study. Anesthesiology.

[14] Levitt JE, Bedi H, Calfee CS (2009). Identification of early acute lung injury at initial evaluation in an acute care setting prior to the onset of respiratory failure. Chest.

[15] Levitt JE, Calfee CS, Goldstein BA (2013). Early acute lung injury: criteria for identifying lung injury prior to the need for positive pressure ventilation*. Crit Care Med.

[16] National Heart, Lung, and Blood Institute PETAL Clinical Trials Network (2019). Early High-Dose Vitamin D3 for Critically Ill, Vitamin D–Deficient Patients. N Engl J Med.

[17] https://www.biolincc.nhlbi.nih.gov/studies/petal_violet/.

[18] The ARDS Definition Task Force (2012). Acute respiratory distress syndrome: the Berlin definition. JAMA.

[19] Peterson SM, Harbertson CA, Scheulen JJ (2019). Trends and characterization of academic emergency department patient visits: a five-year review. Acad Emerg Med.

[20] Singer AJ, Thode HC, Viccellio P (2011). The association between length of emergency department boarding and mortality. Acad Emerg Med.

[21] Mohr NM, Wessman BT, Bassin B (2020). Boarding of critically ill patients in the emergency department. Crit Care Med.

[22] Kim BK, Kim S, Kim CY (2021). Predictive role of lung injury prediction score in the development of acute respiratory distress syndrome in Korea. Yonsei Med J.

