# Supplementary material for: External Validation of a Novel Lung Injury Prevention Score for the Emergency Department
Source: West J Emerg Med. 2025 Dec 19;27(1):146–51. doi: 10.5811/westjem.41994 (PMC12815506; doi:10.5811/westjem.41994)
Supplement: Supplementary file 1 [file wjem-27-146-s001.docx]

**Supplementary Appendix**

**Table S1.** The Berlin Definition of ARDS


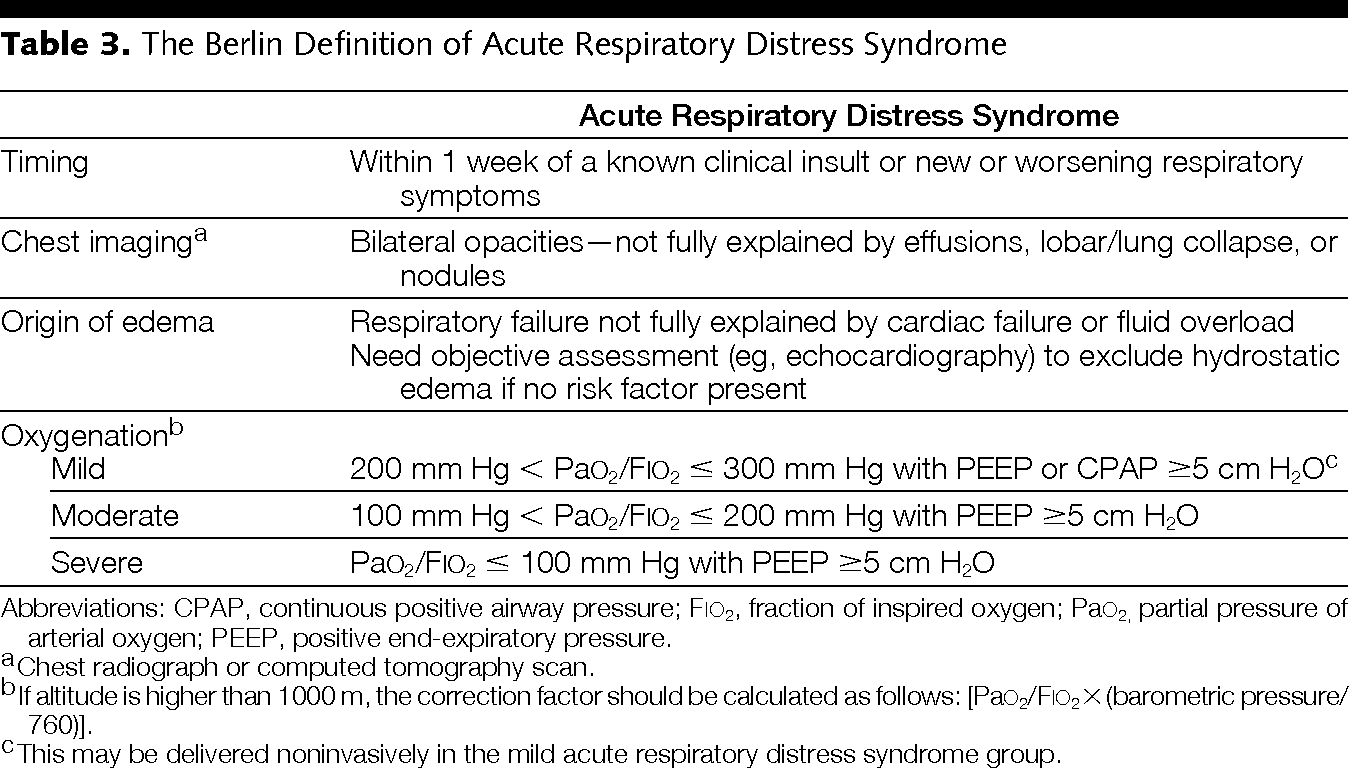


The ARDS Definition Task Force. Acute Respiratory Distress Syndrome: The Berlin Definition. *JAMA*. 2012;307(23):2526-2533.

^a^Chest radiograph or computed tomography scan.

^b^If altitude is higher than 1000m, the correction factor should be calculated as follows: [PaO_2_/FiO_2_ x (barometric pressure/760)].

^c^This may be delivered noninvasively in the mild acute respiratory distress syndrome group.

*CPAP,* continuous airway pressure; *FiO_2_,* fraction of inspired oxygen; *PaO_2_,* partial pressure of arterial oxygen; *PEEP,* positive end-expiratory.

**Table S2.** EDLIPS variables and associated points.

| Predispositions | EDLIPS Points |
| --- | --- |
| Male gender | 1 |
| Aspiration | 2 |
| Pneumonia | 1 |
| Sepsis | 1 |
| Shock | 2 |
| Lung contusion | 1 |
| Smoke inhalation | 1.5 |
| Long bone fractures | 2 |
| Brain injury | 2 |
| Cardiac surgery | 5 |
| Aortic surgery | 5 |
| Spine surgery | 5 |
| Acute abdomen | 2.5 |
| Risk Modifiers |  |
| Diabetes mellitus | -0.5 |
| Cirrhosis | 1 |
| Chemotherapy | 2 |
| Obesity (BMI >30) | 1.5 |
| Acidosis (pH <7.35) | 2 |
| FiO₂ > 0.35 (>4 L/min) | 2 |
| Albumin <3.5 | 1.5 |
| SpO₂ < 95% | 1.5 |


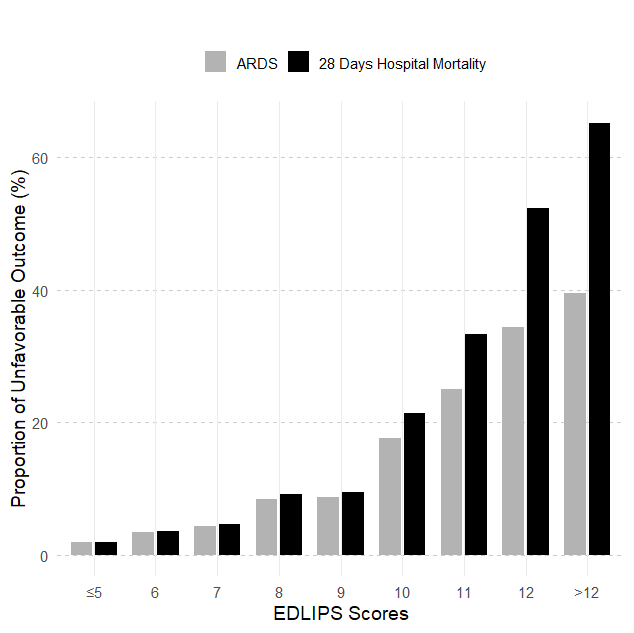


**Figure S1.** Frequency of ARDS development and hospital mortality according to EDLIPS values (n=1270).
